# Factors influencing osteoradionecrosis progression during hyperbaric oxygen therapy: A case study

**DOI:** 10.12688/f1000research.155112.2

**Published:** 2025-01-02

**Authors:** Sameh Mezri, Chaima Zitouni, Khadija Bahrini, Mounir Haggui, Wiem Boughzala, Hedi Gharsallah

**Affiliations:** 1 University of Tunis El Manar, Rommana, Tunis, 1068, Tunisia; 2 ENT department, Military hospital of Tunis, Montfleury, Tunis, 1008, Tunisia; 3 Research Unit UR17DN05, Military hospital of Tunis, Montfleury, Tunis, 1008, Tunisia; 4 Emergency department, Military hospital of Tunis, Montfleury, Tunis, 1008, Tunisia; 5 Department of hyperbaric oxygen therapy, Military Hospital of Tunis, Montfleury, Tunis, 1008, Tunisia

**Keywords:** Osteoradionecrosis, Radiotherapy, Hyperbaric oxygen therapy, Head and neck

## Abstract

**Introduction:**

Although relatively uncommon, osteoradionecrosis (ORN) remains a serious complication following radiotherapy. Various therapeutic approaches, including hyperbaric oxygen therapy (HBOT), are utilized in managing ORN. This study aims to evaluate the role of HBOT in ORN management and to identify predictive factors influencing the evolution of head and neck ORN after HBOT.

**Methods:**

This retrospective study includes 46 patients who received HBOT for head and neck ORN between 2017 and 2020. The patients were divided into two groups: Group 1 (n=36) included those with regression or stabilization of ORN, while Group 2 (n=10) comprised patients with worsening lesions. We performed a statistical study in order to identify factors influencing ORN progression under treatment.

**Results:**

ORN affected the mandible in 93.5% of patients, the maxilla in 2 cases, and the skull base in 4 cases. All patients received HBOT, with an average of 44.65 sessions. Pre-operative HBOT was administered in 17% of cases, and post-operative HBOT was given in 42% of cases. After at least 20 sessions, ORN regressed in 33% of cases, stabilized in 45%, and worsened in 22%.

Analysis of factors influencing ORN progression on the univariate study revealed significant associations with high blood pressure (p=0.046), larger tumor size (p=0.004), advanced tumor stages (p=0.048), mean radiation dose (p=0.002), delays between dental care and radiotherapy (p=0.045), and the location of ORN within the mandible (p=0.049). Additionally, the number of HBOT sessions significantly affected ORN evolution, with more sessions correlating with better outcomes (p=0.001). In the multivariate analysis, variables such as the average interval between dental care and radiotherapy (p=0.043) as well as the number of HBOT sessions (p=0.040) emerged as significant influencers of ORN evolution.

**Conclusion:**

Our study provides valuable insights into the management of ORN by identifying key predictors that influence the post-therapeutic evolution of head and neck ORN after HBOT.

## Introduction

Head and neck cancers rank seventh in the world, with an incidence of 890,000 new cases. ^1^

Their management typically involves surgery, radiotherapy (RT), and chemotherapy. Despite advancements in techniques, external RT often leads to complications, including osteoradionecrosis (ORN).

ORN is defined as progressive bone destruction, occurring spontaneously or following trauma, with mucosal ulceration exposing irradiated bone, persisting for 3 to 6 months without healing, excluding tumor recurrence. ^2^

Although relatively uncommon, ORN remains a serious complication, significantly affecting patients’ quality of life often leading to chronic pain, infections, and functional impairments in the affected areas. ^3^

The pathophysiology of ORN remains poorly understood, despite extensive research. It is believed to result from the interplay between radiation-induced damage to the vasculature, impaired healing, and an inflammatory response. However, there is still no clear consensus on the exact mechanisms behind its development. ^4^

Various therapeutic approaches are utilized in the treatment of ORN. They include antibiotics, hyperbaric oxygen therapy (HBOT), the PENTOCLO protocol, and conservative surgery. Among these, HBOT has garnered significant attention due to its potential to enhance tissue healing and reduce bone damage by improving oxygen supply to irradiated tissues. ^5^

Several studies have evaluated the role of HBOT in managing ORN, with varying results. However, the optimal treatment protocol, including the duration and frequency of HBOT sessions, remains a topic of debate. Moreover, there is limited research identifying predictive factors that influence the outcomes of ORN treatment, particularly concerning the progression of the condition following HBOT. While some studies have shown promising results, the role of HBOT in combination with other treatments and its impact on long-term outcomes remains underexplored.

Our study aims to evaluate the role of HBOT in ORN management and describe predictive factors influencing post-therapeutic evolution of head and neck ORN.

By addressing the current gaps in knowledge, we hope to provide insights that could lead to more effective and personalized treatment strategies for ORN patients.

## Methods

### The cohort study

This is a retrospective study conducted at the HBOT Department of the Military Hospital of Tunis over a period of 4 years, from January 1, 2017, to December 31, 2020. The study included patients who met the following criteria:

- Treated with HBOT for head and neck ORN
- Completed at least 20 HBOT sessions
- Had a complete medical record
- Were available for follow-up evaluations for at least six months after the end of the therapy

All cases with recurrent loco-regional tumor and who received discontinued or irregular HBOT sessions were excluded from this study.

For this study, we developed a specialized data collection tool called a case report form (CRF). This form was used to document various aspects of each patient’s case, including their demographics, clinical examination, laboratory results, imaging, treatment, and follow-up.

ORN was classified into four grades according to the LENT/SOMA (Late Effects of Normal Tissues/Subjective Objective Management Analytic) classification. ^6^

Grade 1: Normal bone appearance or debatable modifications

Grade 2: Bone lysis or condensation

Grade 3: Bone sequestration

Grade 4: Bone fracture

### OHB protocol

HBOT sessions were conducted in a multiplace hyperbaric chambers type HAUX-STARMED 2400. The HBOT protocol included 3 steps:

➢ Step 1: Compression phase: Around 0.1 ATA (Atmosphere Absolute) and the Compression rate was adjusted based on patient’s tolerance.
➢ Step 2: Plateau phase: in this phase patients received 100% oxygen using facial mask for 60 and or 90 minutes with 5-minute air breaks every 25 minutes.
➢ Step 3: decompression phase: this step is slow typically around 0.1 ATA/min.

Patients were followed up during and following HBOT therapy. Clinical and imaging evolution of injuries was recorded.

Radiological examinations, if needed, were performed after at least 40 HBOT sessions. However, if the hyperbaric medicine specialist noticed any signs of worsening condition after 20 HBOT sessions, CT scans were ordered earlier. In our study, only CT was used for radiological control.

ORN injuries were assessed clinically and by imaging following HBOT treatment. Thereby lesion evolution was classified into three categories:

- Regression: Clinically and/or radiologically confirmed the disappearance of functional and radiological signs
- Stabilization: It involves partial healing of the ORN with no progression of bone necrosis. Functional signs are moderate.
- Worsening or Aggravation: Progressive worsening of the ORN characterized by the persistence of functional and radiological signs. There is no bone healing and the ORN was progressed to a more severe stage.

### Statistical analysis

**Figure 1.**
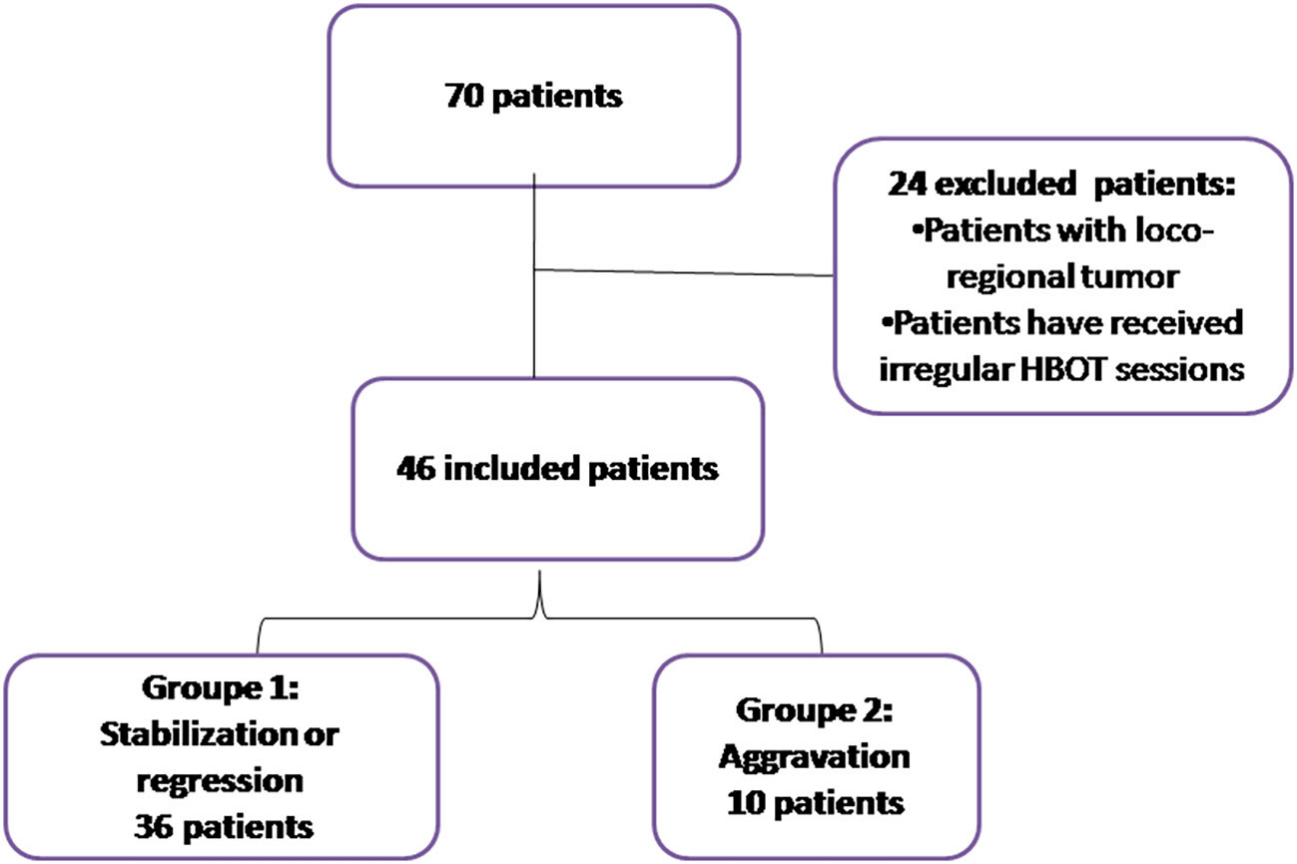
Diagram of patient recruitment, exclusions, and study groups.

Statistical analyses were performed using SPSS version 22.0. Categorical variables were reported as percentages, and their significance was assessed using Fisher’s exact test or chi-square tests. Quantitative variables were presented as mean (standard deviation) or median ± interquartile range (IQR25%, 75%). Normality was tested using the Kolmogorov-Smirnov test. An unpaired t-test was used to compare two groups when continuous variables were normally distributed. For continuous variables that were not normally distributed, a two-tailed unpaired Mann-Whitney U test was applied. Both univariate and multivariate analyses were conducted to evaluate the effectiveness of HBOT on ORN patients. The cases were divided into two groups: Group 1 (n=36) included patients with regression or stabilization, and Group 2 (n=10) included patients with worsening signs of injury after HBOT ( Figure 1). Differences were considered significant if p ≤ 0.05.

### Ethics statement

This study was approved by the Ethics Committee of the Military Hospital of Tunis, under decision number 81/2024/CLPP, dated July 22, 2024. The study was conducted in accordance with the ethical principles outlined in the Declaration of Helsinki ( https://www.wma.net/policies-post/wma-declaration-of-helsinki-ethical-principles-for-medical-research-involving-human-subjects/).

## Results

### Demographic characteristics of the cohort study ( Table 4)

This study included 46 patients. The average age of patients was 58 ± 13.9 years, with the majority of patients (61%) aged between 31 and 65 years old. Fifty-two percent were female, and 48% were male. Two-thirds of the patients had diabetes and 59% were smokers.

### Radiotherapy treatment

All patients had undergone RT for head and neck malignancies, primarily nasopharyngeal carcinoma (67%) ( Table 1).

**Table 1.** Tumor histological type and location.

Tumor histological type and location	Number of patients
Undifferentiated carcinoma of nasopharyngeal type (UCNT)	31
Squamous cell carcinoma of the mandible	2
Gingival squamous cell carcinoma	1
Squamous cell carcinoma of the palate	1
Squamous cell carcinoma of the tonsil	1
Lymphoma with tonsillar localization	1
Squamous cell carcinoma of the tongue	1
Mucoepidermoid carcinoma of the parotid gland	1
Squamous cell carcinoma of the external auditory canal	1
Cutaneous squamous cell carcinoma	1
Breast ductal carcinoma with metastatic cervical lymph nodes	5

The mean tumor size was 29.67 mm.

RT was conventional in 40 cases, cobalt-60 2D dimensional in two cases, three-dimensional in one case, and Intensity-Modulated Radiation Therapy (IMRT) in three cases; with a mean radiation dose of 48.7 Gy. The association with chemotherapy was noted in thirty cases, accounting for 65% of the cases.

Prior to radiotherapy, all patients received comprehensive dental care, including professional cleaning and fluoride treatments. Among these patients, 67% required tooth extractions. Twelve patients used the custom fluoride tray correctly. Patients were also educated on proper oral hygiene practices during and after radiation therapy, emphasizing brushing techniques, fluoride toothpaste, and the importance of regular dental follow-ups. The interval between dental care and the start of RT ranged from one month to 48 months, with a median of two months.

### Osteoradionecrosis diagnosis

ORN typically occurred due to dental extractions (83% of cases) or surgical procedures (15%). The median time from RT to ORN onset was 6 years, while from dental care to ORN onset was 36 months.

Common clinical symptoms included pain (100% of patients) and mastication difficulties (98%). Trismus was present in all patients, with varying degrees of severity. Dental mobility was observed in 33% of cases.

**Figure 2.**
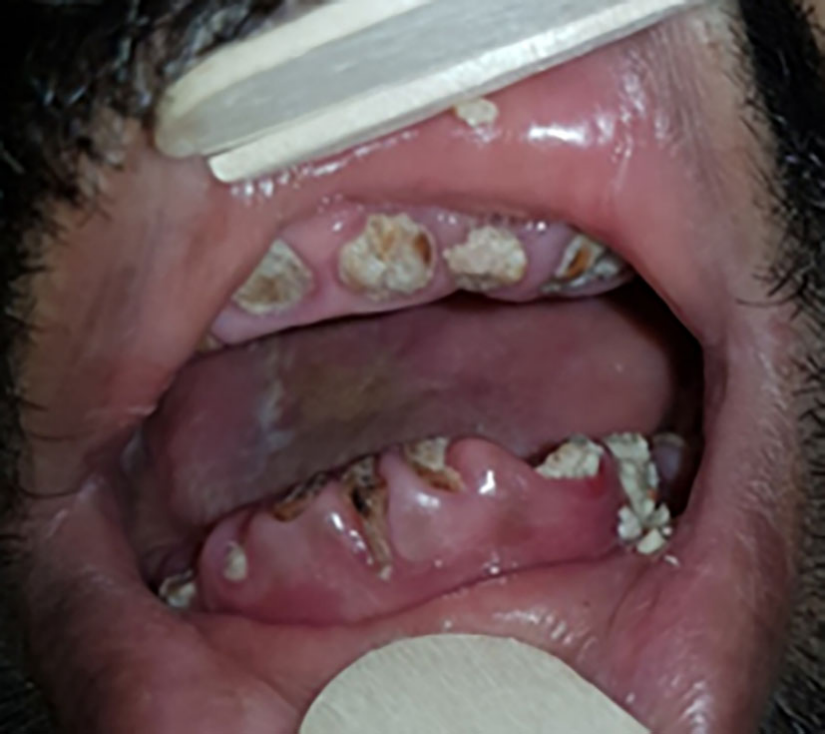
Exposure of the mandibular bone.

**Figure 3.**
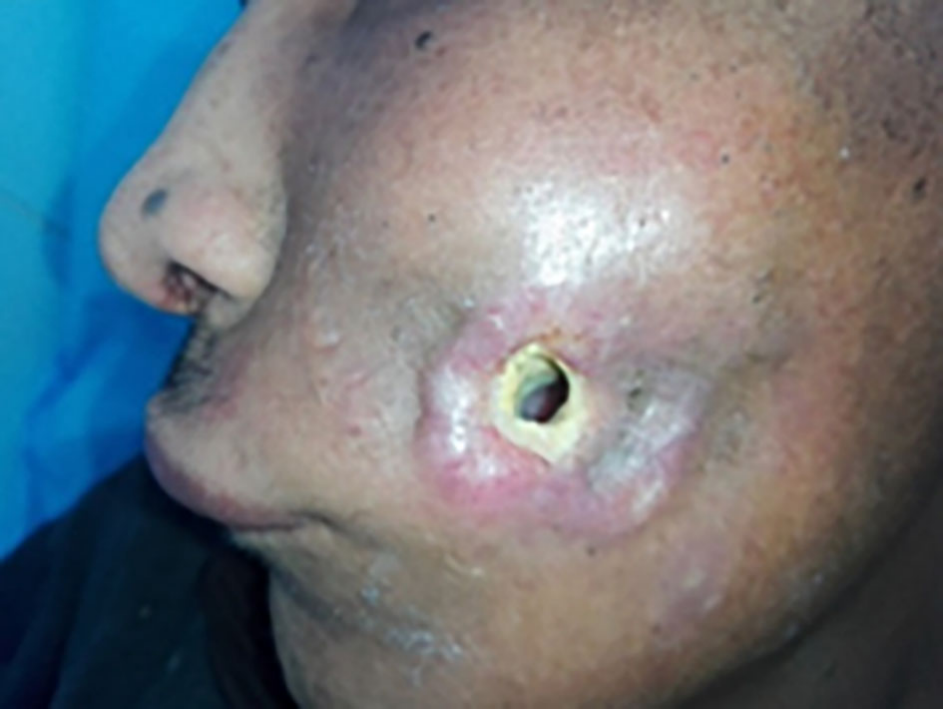
Cutaneous fistula in osteoradionecrosis.

We also noted the presence of bone exposure ( Figure 2) in 6 cases, bone sequestration in 15 cases, or bone fracture in 9 cases. The presence of a cutaneous fistula ( Figure 3) was noted in 39% of cases. In four cases, there was pus discharge through the fistula.

We performed panoramic radiograph (76%), cone beam imaging (11%), computed tomography (CT scan) (80%), and magnetic resonance imaging (MRI) (28%).

Imaging revealed the following grades of ORN according to the LENT/SOMA classification: Grade 2 (43% of cases), Grade 3 (37%), and Grade 4 (20%).

**Figure 4.**
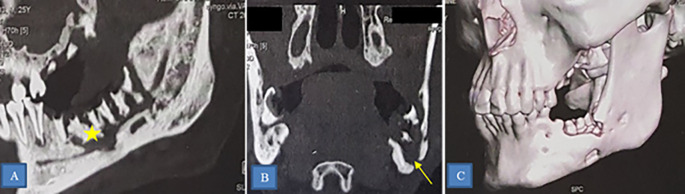
Osteoradionecrosis of the left mandible. CT scan showing osteoradionecrosis of the horizontal branch of the left mandible complicated by sequestra (star) and mandibulo-cutaneous fistula (yellow arrow). A: Sagittal section. B: Frontal section. C: 3D reconstruction.

**Figure 5.**
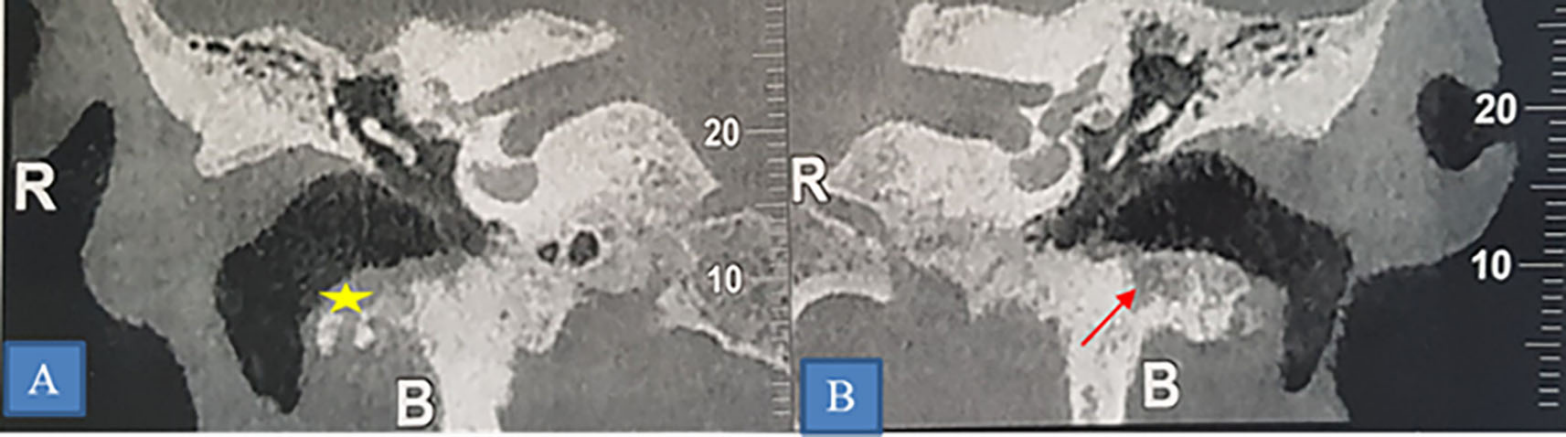
Osteoradionecrosis of the temporal bone. A and B: Coronal section showing of a CT-scan showing bilateral lytic erosion of the tympanic bone (star) and thickening of the walls of the external auditory canal.

ORN was predominantly mandibular (40 patients), affecting various regions such as the horizontal branch (27 patients) ( Figure 4), mandibular angle (10 patients), and ascending branch (3 patients). Other locations included the maxilla (2 cases) and the skull base (4 cases) ( Figure 5).

The anatomopathological examination was performed in one case due to delayed alveolar consolidation and diagnostic doubt for differential diagnosis. It concluded with fibro-inflammatory chronic scarring remodeling, displaying features of ORN without specific granuloma or signs of malignancy.

A bacteriological examination with culture was performed in four cases, yielding negative results in three cases and identifying polymicrobial flora in one case.

### Treatment of osteoradionecrosis

The therapeutic management of ORN included topical antibacterial mouthwash prescribed to all patients. All patients received analgesic treatment based on their pain intensity. Antibiotic therapy was initiated in 89% of cases. In all cases, it was empirical. In about 70% of cases, the treatment was primarily based on amoxicillin and clavulanic acid (89%). The average duration of the treatment was 6 weeks.

Surgical treatment was performed in 36 patients, predominantly involving sequestrectomy (94% of cases). Mandibulectomy with mandibular reconstruction using flaps was conducted in 6% of cases.

HBOT was provided to all patients, with a mean of 44.65 sessions. HBOT sessions preceded ORN surgery in 17% of cases and were indicated after surgery in 42% of cases.

Complications of HBOT were observed in two patients, including middle ear barotrauma, necessitating transient discontinuation of therapy.

**Figure 6.**
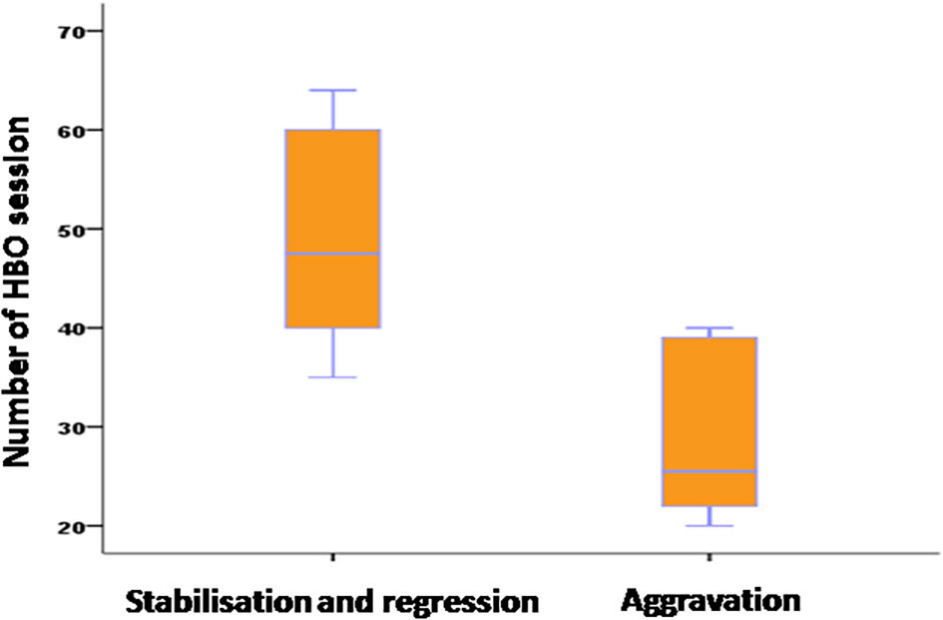
Evolution of osteoradionecrosis based on the number of hyperbaric oxygen therapy sessions.

Evolution of ORN lesions was evaluated clinically after 20 HBOT sessions and radiologically after 40 sessions. Results showed regression in 33% of cases, stabilization in 45%, and progression or aggravation in 22% ( Figure 6).

### Risk factors associated with osteoradionecrosis aggravation

In the univariate study ( Table 2), age and sex did not show statistical significance in influencing ORN progression. However, among comorbidities, hypertension was significantly associated with worsened ORN evolution.

**Table 2.** Univariate study of factors influencing the post-therapeutic evolution of osteoradionecrosis.

		1st group (%)	2 ^nd^ group (%)	p
Age groups	< 31	1 (3)	2 (20)	0.148
	[31- 65]	21 (58)	5 (50)	
	≥65	14 (39)	3 (30)	
Gender	Male	19 (53)	3 (30)	0.202
	Female	17 (47)	7 (70)	
Diabetes	Yes	23 (64)	7 (70)	0.720
	No	13 (36)	3 (30)	
High blood pressure	Yes	20 (56)	2 (20)	0.046
	No	16 (44)	8 (80)	
Smoking	Yes	20 (56)	5 (50)	0.755
	No	16 (44)	5 (50)	
Tumor site	Nasopharynx	22	9	0.997
	Mouth	8	0	
	Breasts	4	1	
	External auditory canal	1	0	
	Skin	1	0	
Mean tumor size		27.9 mm	36 mm	0.004
Tumor stage	T2	13 (95)	1 (11)	0.048
	T3	8 (36)	7 (78)	
	T4	1 (4)	1 (11)	
Type of RT	Conventional RT	30 (83)	10 (100)	0.590
	IMRT	3 (8)	0	
	3D RT	1 (3)	0	
	2D cobalt-60 RT	2 (6)	0	
Mean radiation dose		42	68	0.021
Chemotherapy	Yes	22 (61)	8 (80)	0.267
	No	14 (39)	2 (20)	
The average interval between dental care (before RT) and RT		5.46 months	1.6 months	0.045
Dental extraction after RT	Yes	29 (81)	9 (90)	0.486
	No	7 (19)	1 (10)	
Location of the ORN	Horizontal branch of the mandible	22 (74)	5 (50)	0.049
	Mandibular angle	5 (17)	5 (50)	
	Ascending ramus of the mandible	3 (10)		
Radiological signs	Bone lysis	15 (42)	5 (50)	0.686
	Bone sequestrum	13 (36)	4 (40)	
	Bone fracture	8 (22)	1 (10)	
Antibiotic therapy	Yes	32(89)	9(90)	0.920
	No	4(11)	1(10)	
Average number of HBOT sessions		49.11	28.6	0.001
Surgery after RT	Yes	7 (20)	1 (10)	0.466
	No	28 (80)	9 (90)	
Type of surgery	Sequestrectomy	28 (93)	6 (100)	0.515
	Mandibular reconstruction with flap	2 (7)	0	
Interval between HBOT and surgery	Before	4	2	0.359
	Simultaneously	13	2	
	After	13	2	

RT: Radiotherapy; ORN: Osteradionecrosis; IMRT: Intensity-Modulated Radiation Therapy; HBOT: Hyperbaric Oxygen Therapy.

Our study also revealed that larger tumor size and advanced tumor stages correlated with worse outcomes, while tumor location did not statistically influence ORN evolution.

The type of radiotherapy did not statistically influence the progression of ORN lesions, nor did its association with chemotherapy. Higher radiation therapy doses statistically influenced ORN evolution.

Delays between dental care (before RT) and RT were associated with worse ORN evolution, while dental care after RT did not significantly influence it.

The location of ORN within the mandible significantly influenced lesion evolution, but radiological signs did not.

The use of antibiotics did not statistically influence the progression of ORN lesions. HBOT significantly influenced ORN evolution, with more sessions correlating with better outcomes. We observed that the progression of lesions in patients who underwent surgery was not influenced by the timing of surgery, whether it was before, concurrent with, or after HBOT therapy. The type of surgical treatment did not statistically influence lesion evolution.

In the multivariate analysis ( Table 3), variables such as the average interval between dental care (before RT) and RT and the number of HBOT sessions emerged as significant influencers of ORN evolution. Specifically, extending the delay between dental care and RT by one month correlated with a sevenfold enhancement in ORN lesion evolution. Furthermore, each additional ten HBOT sessions were linked with a tenfold improvement in ORN lesion evolution.

**Table 3.** Multivariate study of factors influencing the post-therapeutic evolution of osteoradionecrosis.

	P	OR	95% confidence interval for EXP(B)	
			Lower value	Upper value
High blood pressure	0.623	1.803	0.172	18.923
Tumor mean size	0.778	1.022	0.878	1.189
Mean radiation dose	0.193	1.055	0.973	1.145
The average interval between dental care before RT and RT	0.043	7.055	1.067	46.649
Dental extraction after RT	0.121	6.159	0.618	61.371
Location of the ORN	0.909			
Average number of HBO sessions	0.040	1.041	1.021	1.062

RT: Radiotherapy; ORN: Osteoradionecrosis; HBO: Hyperbaric Oxygen Therapy.

**Table 4.** Demographic and clinical characteristics of study participants.

Characteristic	Total (n = 46)	%
**Mean Age (years)**	58 ± 13.9	-
**Gender** - Female - Male	24 22	52% 48%
**Diabetes**	30	65%
**Smoking**	27	59%
**High blood pressure**	22	48%

## Discussion

In 1973, Mainous et al. ^7^ first proposed HBOT as a treatment for ORN. Subsequent studies have highlighted the significant benefits of HBOT in managing ORN, primarily due to its ability to deliver hyper-concentrated oxygen levels, up to 20 times higher than normal conditions. HBOT facilitates increased oxygen diffusion in hypoxic tissues, stimulates osteogenesis and mucosal tissue epithelialization, promotes collagen synthesis and osteoblast proliferation in irradiated tissues, enhances the expression of Vascular Endothelial Growth Factor (VEGF) leading to angiogenesis, improves tissue oxygen perfusion, and exerts a bactericidal and bacteriostatic effect against various pathogens. These mechanisms collectively underscore the therapeutic potential of HBOT in the management of ORN. ^8^

Several studies have reported healing rates ranging from zero to 100%. ^9– 14^ However, comparing these results is challenging due to the variability of protocols applied and the variability in case indications (varying severity of ORN cases).

Many authors have demonstrated improvement in ORN with HBOT. ^15– 17^

Besides its therapeutic effect on ORN lesions, HBOT can help improve patients’ quality of life by reducing pain, promoting wound healing, and lowering the risk of infectious complications. ^18^

However, Annane et al. ^19^ halted their trial due to a significantly better healing rate in the placebo group (32%) compared to the HBOT group (19%). The main criticisms of this trial were the monomodal use of HBOT in treating his patients and the lack of strict and clear criteria for defining ORN and its severity.

According to recommendations established by medical organizations such as the Undersea and Hyperbaric Medical Society (UHMS) ^18^ and the Tenth European Consensus Conference on Hyperbaric Medicine, ^20^ HBOT is recommended for treating symptomatic ORN cases or as an adjunct treatment to surgical intervention in order to enhance wound healing, reduce infection risk, and promote tissue healing after reconstructive or debridement surgery.

According to the UHMS, to achieve these effects, the recommended pressure should be equal to or greater than 1.4 atmospheres (atm). However, all current indications approved by the UHMS require patients to breathe nearly 100% oxygen in a pressurized chamber at a minimum pressure of 2 ATA. ^18^ However, results appear controversial and inconclusive. Thus, at the limit of our literature search, only one randomized clinical trial has been published, with other studies mainly consisting of cohort studies of varying quality.

A study by Annane et al. ^19^ examined the efficacy of HBOT in the treatment of ORN and found that patients who received a higher number of HBOT sessions had higher healing rates.

A meta-analysis by Bennett et al. (2016) ^21^ also examined the results of several studies on HBOT in the treatment of ORN. They found that healing rates were higher in patients who received a higher number of HBOT sessions, although the results varied depending on the healing criteria used in the different studies.

Marx et al. ^22^ reported healing rates of up to 85% in patients who received more than 20 HBOT sessions. This was applicable to our study where a minimum of 20 sessions was indicated for our patients, although.patient compliance during this extended treatment plays a critical role in achieving optimal outcomes.

The combination of HBOT and surgical treatment has been addressed in the literature. Several authors have reported ORN healing rates of 15% to 45% with HBOT alone and 20% to 90% when HBOT was combined with surgery. ^23– 25^ However, these studies lack precision with somewhat heterogeneous groups.

In a recently published multicenter randomized trial in 2021, Forner et al. ^26^ reported a significantly better healing rate in the HBOT-surgery group (surgical treatment preceded by 30 HBOT sessions and followed by 10 HBOT sessions) at 70% (21/30) compared to 51% (18/35) in the surgery-only group. HBOT was associated with improved healing rates, regardless of ORN severity. It also reduced the severity of xerostomia and dysphagia and improved total unstimulated salivary flow. However, despite the most supported multimodal approach to ORN management being the combination of HBOT and surgery, due to the divergent conclusions of the literature, establishing standardized protocols for HBOT use in parallel with surgery seems compromised.

Broad-spectrum antibiotics such as penicillins, metronidazole, and clindamycin are commonly used in ORN due to their effectiveness against anaerobic bacteria often involved. ^27^ Aas et al. ^28^ used culture-independent molecular techniques to identify 59 bacterial species in ORN specimens, with anaerobes predominating and 27% of the bacteria being uncultivable, highlighting the complexity of microbial involvement in ORN and the need for comprehensive clinical management. There is no consensus on the duration of antibiotic treatment for ORN. ^27^

HBOT demonstrates a synergistic effect when combined with various antibiotics. It exerts bactericidal and bacteriostatic effects on anaerobic bacteria by promoting the generation of free oxygen radicals. It also enhances the bacterial-killing efficacy of leukocytes in hypoxic wounds by elevating tissue oxygen levels. ^29^

The evolution of ORN under treatment can be influenced by several factors.

Oh et al., ^14^ studied factors influencing ORN evolution, collecting 114 patients treated for ORN over a 16-year period. They were divided into two groups: group 1 of 47 patients treated with conservative treatment (sequestrectomy, debridement, and/or HBOT) and group 2 of 67 patients treated with immediate intervention or after failure of conservative treatment.

Patients whose ORN was associated with an early-stage tumor or extraction before irradiation responded favorably to conservative treatment. However, patients with advanced primary tumors, who continued to smoke and drink after RT, who received palliative RT or a radiation dose exceeding 60Gy, and who had oro-cutaneous fistulas, pathological fractures, swelling, or trismus responded poorly to conservative treatment. In these latter cases, radical resection of the affected tissue proved useful.

Smoking induces vasoconstriction and oxidative damage, reducing oxygen supply to irradiated tissues and impairing healing. ^30^ Similarly, alcohol contributes to systemic inflammation and immune suppression. ^31^

Hypertension can exacerbate ORN due to its association with vascular dysfunction, which reduces microvascular density and perfusion in irradiated tissues. This condition worsens hypoxia, delaying healing and promoting fibrosis. Hypertension-induced chronic inflammation and oxidative stress further compromise tissue repair mechanisms. ^32^

Larger tumors and advanced staging necessitate higher doses of RT, leading to greater damage to surrounding healthy tissues.

Mandibular bone, in particular, is vulnerable to radiation-induced hypoxia and fibrosis due to dense cortical bone and limited vascular supply; which are primary drivers of ORN progression. ^33^

The onset time of ORN relative to RT is a factor influencing the evolution of ORN lesions. This notion was supported by Oh et al., ^14^ who reported that patients whose ORN occurred within 12 months after RT had a higher resolution rate with conservative treatment than patients whose ORN occurred after 12 months.

Beumer et al. ^34^ reported that ORN occurring after a dose of 70 Gy did not systematically respond to conservative treatment measures, thus requiring non-conservative treatment. At these levels, there is significant cytotoxicity to osteoblasts and endothelial cells, leading to impaired bone remodeling and microvascular obliteration. ^35^ Also, the overall dose and the distribution of that dose across the mandible are key factors. While IMRT can reduce the maximum dose to the mandible, the risk of ORN is still significantly influenced by the total dose delivered to specific mandibular regions. Doses in the range of 35–73 Gy, rather than a single point dose, are crucial in determining the likelihood of ORN development. ^36^

In the same study, the authors reported that ORN occurring due to irritation from dental prostheses or extraction before irradiation respond more effectively to conservative treatment than ORN occurring due to dental disease, either spontaneously or in association with post-irradiation extraction. The latter often requires a radical approach; this result was refuted by Oh et al. ^14^

Tooth extractions should be performed based on pre-radiotherapy dental assessments to remove infected, non-restorable, or periodontally compromised teeth, as these are at higher risk for infection and subsequent ORN. ^37^ A tooth extraction should ideally occur at least two weeks before radiotherapy. ^33^

In a retrospective study conducted by De Felice ^38^ published in 2016, comparing resolved ORN and unresolved ORN, no factor was identified as influencing the resolution or progression of ORN during logistic regression.

In our study, the evolution of ORN lesions was influenced by the presence of hypertension, tumor size, T and N stages, RT dose, dental care delays relative to RT, ORN location within the mandible, and HBOT sessions. During logistic regression, only delays in dental care relative to RT would influence the evolution of ORN lesions.

Despite being the only study conducted in Tunisia on this topic, this research has several limitations that should be acknowledged. First, as a retrospective study, it has missing clinical data. Second, evaluating the efficacy of HBOT ideally requires a control group, which could potentially be addressed with a matched sample from another center. Third, the limited number of cases and the heterogeneity among patients in terms of therapeutic modalities prevent definitive conclusions. Additionally, the variability in radiation doses received by different parts of the jaw, depending on tumor histology and location, may also influence the outcomes. For these reasons, further research is needed to gain a deeper understanding of the therapeutic strategies for ORN tailored to individual patient profiles.

Despite these limitations, our study offers valuable insights into the management of ORN within the Tunisian context and provides a foundation for future, more comprehensive investigations.

## Conclusion

In conclusion, our study yields crucial insights into the management of ORN by identifying significant predictors influencing post-therapeutic evolution of head and neck ORN. These findings underscore the multifactorial nature of ORN progression, implicating patient characteristics, tumor attributes, and treatment modalities.

Notably, advanced T stage, higher RT doses, and shorter delays between dental care and RT initiation were associated with worsened ORN evolution. Conversely, longer delays between dental care and RT initiation, mandibular horizontal branch localization and increased number of HBOT sessions were associated with improved lesion evolution. Logistic regression identified delay between dental care and RT initiation and number of HBOT sessions as independent factors influencing lesion evolution.

These findings highlight the importance of timely intervention and comprehensive treatment strategies in reducing ORN progression and improving patient outcomes. Moreover, our study underscores the potential of HBOT as a valuable adjunctive treatment option in ORN management. However, the long-term effects of HBOT, including potential complications such as barotrauma, oxygen toxicity, and claustrophobia, must be carefully considered in treatment planning. Further research is warranted to validate these findings and develop targeted therapeutic approaches tailored to individual patient profiles.

## Consent for publication

All photographs included in this manuscript have been published with the explicit written consent of the individuals depicted. Written consent to publish these images was obtained from the participants prior to submission of the manuscript. Any identifiable features, such as personal details or medical record numbers, have been removed to ensure privacy and confidentiality.

## Ethical and consent

This study was approved by the Ethics Committee of the Military Hospital of Tunis, under decision number 81/2024/CLPP, dated July 22, 2024. The study was conducted in accordance with the ethical principles outlined in the Declaration of Helsinki ( https://www.wma.net/policies-post/wma-declaration-of-helsinki-ethical-principles-for-medical-research-involving-human-subjects/).

The research was conducted ethically, with all study procedures being performed in accordance with the requirements of the World Medical Association’s Declaration of Helsinki.

Written informed consent for publication of their clinical details and/or clinical images was obtained from the patient/parent/guardian/relative of the patient.

## Author contributions

Dr Sameh Mezri: Conceptualization, Methodology

Dr Chaima Zitouni: Writing – Original Draft Preparation – Review and editing

Dr Khadija Bahrini: Formal Analysis

Dr Mounir Haggui: Supervision

Dr Wiem Boughzala: Investigation

Dr Hedi Gharsallah: Resources

## Data Availability

The underlying data for this project are available in the Zenodo repository. Zenodo: Factors influencing osteoradionecrosis progression during hyperbaric oxygen therapy: A case study
1.
https://doi.org/10.5281/zenodo.13901089 ^39^
This project contains the following data:
**Case Report Form**
2.
https://doi.org/10.5281/zenodo.13901073 ^40^
This project contains the following data:
**
Participant Information Sheet** Details of license: The data files are published under a Creative Commons Attribution 4.0 International license (CC BY 4.0).

## Software availability statement

The statistical analyses in this study were conducted using SPSS version 22.0 (IBM Corp, Armonk, NY, USA). Since SPSS is proprietary software, a free alternative that can perform similar statistical functions is R ( https://www.r-project.org/), an open-source software environment for statistical computing and graphics.
