# Supplementary material for: Factors influencing osteoradionecrosis progression during hyperbaric oxygen therapy: A case study
Source: F1000Res. 2025 Jan 2;13:1225. Originally published 2024 Oct 14. [Version 2] doi: 10.12688/f1000research.155112.2 (PMC11809644; doi:10.12688/f1000research.155112.2)
Supplement: Peer review history

# Reviewer report: Reviewer response for version 2

**DOI:** 10.5256/f1000research.175997.r355375

**Published:** 2025-01-03

**Authors:** Atsushi Shudo

**Affiliations:** 1 Kishiwada Tokushukai Hospital, Kishiwada, Japan

Dear Authors,

 Your manuscript has been well revised.

 The insightful article would be very useful to clinicians.

 Thank you for your careful revisions.

 Best regards.

Are enough details provided of any physical examination and diagnostic tests, treatment given and outcomes?

Yes

Is the background of the cases’ history and progression described in sufficient detail?

Yes

Is sufficient discussion included of the importance of the findings and their relevance to future understanding of disease processes, diagnosis or treatment?

Yes

Is the conclusion balanced and justified on the basis of the findings?

Yes

Reviewer Expertise:

oral and maxillofacial surgery, oral oncology, jaw deformity, dental infection, osteonecrosis of the jaws, osteoradionecrosis

I confirm that I have read this submission and believe that I have an appropriate level of expertise to confirm that it is of an acceptable scientific standard.

# Reviewer report: Reviewer response for version 1

**DOI:** 10.5256/f1000research.170239.r332802

**Published:** 2024-11-08

**Authors:** Amaliya Amaliya

**Affiliations:** 1 Universitas Padjadjaran, Bandung, West Java, Indonesia

Dear Authors,

 The manuscript you submitted is well-written and well-read, but unfortunately, I have some comments that need to be addressed by you to improve the article.

 1. First of all, I would like to suggest a minor revision to the title. It would be better if the title also mentioned the type of the study. The authors defined the study as retrospective, but I may suggest it as a case-control study. Either it may be like  Factors influencing osteoradionecrosis progression during hyperbaric oxygen therapy: A retrospective study

 or Factors influencing osteoradionecrosis progression during hyperbaric oxygen therapy: A case-control study.

 2. The introduction is too short, the authors only cite 2 articles in there. Please explain the current state of the knowledge, with references. Identify the knowledge gap that you wish to fill with your study.

 3. The result section is lacking demographic data/table of the subjects.

 4. Did the authors adjust the collected data, for example for differences in age and gender?

 5. In the discussion, please explain the mechanism or at least biological plausibility for the factors associated or influencing the ORN progression, for example, hypertension, what is the explanation or biological plausibility.

Are enough details provided of any physical examination and diagnostic tests, treatment given and outcomes?

Yes

Is the background of the cases’ history and progression described in sufficient detail?

Partly

Is sufficient discussion included of the importance of the findings and their relevance to future understanding of disease processes, diagnosis or treatment?

Partly

Is the conclusion balanced and justified on the basis of the findings?

Yes

Reviewer Expertise:

Periodontology, Tobacco Harm Reduction, Nutrition and Periodontal Health

I confirm that I have read this submission and believe that I have an appropriate level of expertise to confirm that it is of an acceptable scientific standard, however I have significant reservations, as outlined above.

# Response

**Published:** 2024-12-13

**Authors:** Chaima zitouni

**Response to Reviewer Report by** **Amaliya Amaliya,**

**Universitas Padjadjaran, Bandung, West Java, Indonesia**

 We sincerely thank Dr. Amaliya for the thoughtful review and valuable comments, and here are our detailed responses to the comments provided:

**1. First of all, I would like to suggest a minor revision to the title. It would be better if the title also mentioned the type of the study. The authors defined the study as retrospective, but I may suggest it as a case-control study. Either it may be like Factors influencing osteoradionecrosis progression during hyperbaric oxygen therapy: A retrospective study or Factors influencing osteoradionecrosis progression during hyperbaric oxygen therapy: A case-control study.**

**Author Response: **The initial title of the manuscript was **"Factors influencing osteoradionecrosis progression during hyperbaric oxygen therapy: A retrospective study"**, but this was rejected by the journal editors. They proposed the revised title, and we followed their recommendation.

**2. The introduction is too short, the authors only cite 2 articles in there. Please explain the current state of the knowledge, with references. Identify the knowledge gap that you wish to fill with your study.**

**Author Response: **We have expanded the introduction to include a detailed explanation of the current state of knowledge with additional references and have clearly identified the knowledge gap our study aims to address.

**3. The result section is lacking demographic data/table of the subjects.**

**Author Response: **I have included a table to address the reviewer’s concern and enhance the clarity of the results section.

**4. Did the authors adjust the collected data, for example for differences in age and gender?**

**Author Response: **We have adjusted the collected data for potential differences in age and gender in our analysis. Regression analysis was applied to account for these factors and ensure that the results are not confounded by demographic variables.

**5. In the discussion, please explain the mechanism or at least biological plausibility for the factors associated or influencing the ORN progression, for example, hypertension, what is the explanation or biological plausibility.**

**Author Response: **We have addressed the concern regarding the biological plausibility of factors influencing the progression of ORN, including hypertension.

# Reviewer report: Reviewer response for version 1

**DOI:** 10.5256/f1000research.170239.r332798

**Published:** 2024-10-31

**Authors:** Busra Yilmaz

**Affiliations:** 1 Istinye University, Istanbul, Turkey

*Comments to Authors*

 This study addresses the effects of hyperbaric oxygen therapy (HBOT) on osteoradionecrosis (ORN) and analyzes the factors influencing the progression of ORN. The study presents significant clinical insights on the subject; however, several critical gaps need to be addressed and supplemented with additional information:

 1. The authors should explain how the clinical and radiological diagnosis of ORN was determined and provide appropriate references.

 2. The criteria for tooth extraction indications should be clarified, with sources cited.

 3. Details on the pre-treatment dental care performed to minimize the risk of ORN should be provided. Specifically, it should be explained what procedures were carried out as part of dental care, including professional cleaning, fluoride treatments, necessary extractions, restorations, and any patient education to promote oral hygiene during and after radiation therapy.

 4. The criteria used for diagnosing radiation-induced trismus must be explained in detail, along with relevant references.

 5. A key parameter in ORN is the specific radiation dose received by the affected region. For instance, the Dmean Vx doses at extraction sites are not reported.

 6. It would be beneficial to discuss the clinical and laboratory factors that can influence ORN development and treatment from all angles. Recently published reviews may provide useful insights.

 7. In Table 1, the tumor histological types and locations are quite heterogeneous, meaning the radiation doses received by different parts of the jaw also vary, which could affect the outcomes. This limitation should be explicitly addressed.

 Long-term follow-up processes in ORN management could be emphasized. In particular, the effects of patient compliance during long-term treatments, such as HBOT, should be discussed.

 8. The long-term effects of HBOT and any potential complications should also be addressed.

Are enough details provided of any physical examination and diagnostic tests, treatment given and outcomes?

Partly

Is the background of the cases’ history and progression described in sufficient detail?

Partly

Is sufficient discussion included of the importance of the findings and their relevance to future understanding of disease processes, diagnosis or treatment?

Partly

Is the conclusion balanced and justified on the basis of the findings?

Yes

Reviewer Expertise:

osteoradionecrosis, radiation induced trismus, head and neck cancers, chemotherapy, radiotherapy, oral management, oral oncology, side effects

I confirm that I have read this submission and believe that I have an appropriate level of expertise to confirm that it is of an acceptable scientific standard, however I have significant reservations, as outlined above.

# Response

**Published:** 2024-12-13

**Authors:** Chaima zitouni

**Response to Reviewer Report by** **Busra Yilmaz,**

**Istinye University, Istanbul, Turkey**

 We sincerely thank Dr. Yilmaz for the thoughtful review and valuable comments, and here are our detailed responses to the comments provided:

1. **The authors should explain how the clinical and radiological diagnosis of ORN was determined and provide appropriate references.**

The diagnosis of ORN in our study was based on both clinical assessment and radiological imaging, as follows:

Clinical Diagnosis: The primary clinical symptoms included pain, mastication difficulties, trismus, dental mobility, and bone exposure. These are typical symptoms of ORN and were observed in the majority of patients. Additionally, the presence of cutaneous fistulas was noted in 39% of cases, which is a key indicator of more advanced ORN.

Radiological Diagnosis: Radiological examinations, including panoramic radiographs, CT scans, and MRI, were used to assess the severity of ORN. CT scans were the primary imaging modality for evaluating ORN progression. In our study, ORN lesions were classified according to the LENT/SOMA grading system

1. **The criteria for tooth extraction indications should be clarified, with sources cited.**

We have clarified the criteria for tooth extraction indications in the discussion section.

1. **Details on the pre-treatment dental care performed to minimize the risk of ORN should be provided. Specifically, it should be explained what procedures were carried out as part of dental care, including professional cleaning, fluoride treatments, necessary extractions, restorations, and any patient education to promote oral hygiene during and after radiation therapy.**

We provided the necessary details about the pre-treatment dental care performed to minimize the risk of ORN.

1. **The criteria used for diagnosing radiation-induced trismus must be explained in detail, along with relevant references.**

The diagnosis of radiation-induced trismus was not part of the objectives of our study, and therefore, we did not include detailed criteria for its diagnosis.

1. **A key parameter in ORN is the specific radiation dose received by the affected region. For instance, the Dmean Vx doses at extraction sites are not reported.**

We did not measure the specific radiation doses, such as the Dmean Vx doses at the extraction sites, in our study. However, we acknowledge the importance of radiation dose as a key parameter in ORN and have included a discussion of its role and relevance in the progression of ORN in the manuscript.

1. **It would be beneficial to discuss the clinical and laboratory factors that can influence ORN development and treatment from all angles. Recently published reviews may provide useful insights.**

In our study, we have made an effort to enrich the discussion by including relevant clinical factors that could influence the development and treatment of ORN.

**7. In Table 1, the tumor histological types and locations are quite heterogeneous, meaning the radiation doses received by different parts of the jaw also vary, which could affect the outcomes. This limitation should be explicitly addressed.**

**Long-term follow-up processes in ORN management could be emphasized. In particular, the effects of patient compliance during long-term treatments, such as HBOT, should be discussed.**

 We have addressed this limitation by acknowledging the variability in radiation doses due to the heterogeneity of tumor histology and locations in the revised discussion.

 We have included a discussion on the importance of long-term follow-up in ORN management, emphasizing that patient compliance during extended treatments, such as HBOT, plays a crucial role in achieving optimal outcomes.

1. **The long-term effects of HBOT and any potential complications should also be addressed.**

We have added information on the long-term effects of HBOT, including potential complications.

# Reviewer report: Reviewer response for version 1

**DOI:** 10.5256/f1000research.170239.r332796

**Published:** 2024-10-25

**Authors:** Atsushi Shudo

**Affiliations:** 1 Kishiwada Tokushukai Hospital, Kishiwada, Japan

Dear Authors,

 Thanks for the chance to review the manuscript entitled “Factors influencing osteoradionecrosis progression during hyperbaric oxygen therapy: A case study”.

 The reviewer is an oral maxillofacial surgeon.

 I read with great interest your paper. I also use HBOT to treat ORN, and found this paper extremely useful.

 Please consider the following points.

 - Since ORN is an infectious osteomyelitis, I suggest a more detailed description regarding antimicrobials should be done. Would it be possible to consider the type of antibiotics and the administration duration? As the authors know, HBOT is believed to enhance the effectiveness of antibiotics.

 - In relation to the above, could you please mention bacterial testing? The presence or absence of anaerobic bacteria seems important in discussing the effectiveness of HBOT.

 Best regards.

Are enough details provided of any physical examination and diagnostic tests, treatment given and outcomes?

Yes

Is the background of the cases’ history and progression described in sufficient detail?

Yes

Is sufficient discussion included of the importance of the findings and their relevance to future understanding of disease processes, diagnosis or treatment?

Yes

Is the conclusion balanced and justified on the basis of the findings?

Yes

Reviewer Expertise:

oral and maxillofacial surgery, oral oncology, jaw deformity, dental infection, osteonecrosis of the jaws, osteoradionecrosis

I confirm that I have read this submission and believe that I have an appropriate level of expertise to confirm that it is of an acceptable scientific standard, however I have significant reservations, as outlined above.

# Response

**Published:** 2024-12-13

**Authors:** Chaima zitouni

**Response to Reviewer Report by** **Atsushi Shudo,**

**Kishiwada Tokushukai Hospital, Kishiwada, Japan**

 We sincerely thank Dr. Atsushi Shudo for the thoughtful review and valuable comments, and here are our detailed responses to the comments provided:

1. **Since ORN is an infectious osteomyelitis, I suggest a more detailed description regarding antimicrobials should be done. Would it be possible to consider the type of antibiotics and the administration duration? As the authors know, HBOT is believed to enhance the effectiveness of antibiotics.**

We have addressed this suggestion by providing a more detailed description of the antimicrobials used, including the types of antibiotics and their administration duration, while also discussing the potential role of HBOT in enhancing antibiotic effectiveness.

  

1. **In relation to the above, could you please mention bacterial testing? The presence or absence of anaerobic bacteria seems important in discussing the effectiveness of HBOT.**

We have included a discussion on bacterial testing, highlighting the importance of anaerobic bacteria in ORN, and emphasized the potential role of HBOT in enhancing the effectiveness of antibiotics against these bacterial profiles.
